# Opioid Use Disorder Induces Oxidative Stress and Inflammation: The Attenuating Effect of Methadone Maintenance Treatment

**Published:** 2018-01

**Authors:** Ali Salarian, Mehri Kadkhodaee, Maryam Zahmatkesh, Behjat Seifi, Enayatollah Bakhshi, Shahin Akhondzadeh, Soheila Adeli, Hassan Askari, Mohammad Arbabi

**Affiliations:** 1 Department of Neuroscience and Addiction Studies, School of Advanced Technologies in Medicine, Tehran University of Medical Sciences, Tehran, Iran.; 2 Department of Physiology, School of Medicine, Tehran University of Medical Sciences, Tehran, Iran.; 3 Research Center of Behavioral and Cognitive Sciences, Tehran University of Medical Sciences, Tehran, Iran.; 4 Department of Biostatistics, University of Social Welfare and Rehabilitation Sciences, Tehran, Iran.; 5 Psychiatric Research Center, Roozbeh Hospital, Tehran University of Medical Sciences, Tehran, Iran.

**Keywords:** *Oxidative Status*, *Opioid Use Disorder*, *Methadone Maintenance Treatment*

## Abstract

**Objective:** Frequent use of opioids produces reactive oxygen species, upregulates inflammatory factors, and contributes to opiate dependence. In this study, we examined perturbations of plasma oxidative and inflammatory markers in patients with opioid use disorder in two phases. In the first phase, we compared the oxidative status in patients with opioid use disorders and in healthy controls; and in the second phase, we examined oxidative changes before and after methadone maintenance treatment.

**Method:** To explore whether oxidative changes were associated with opioid use disorder, we compared plasma oxidative and inflammatory markers in patients with opioid use disorder and in smoking and non-smoking healthy participants. All participants completed measures of catalase (CAT), glutathione (GSH), malondialdehyde (MDA), superoxide dismutase (SOD), matrix metalloproteinase (MMP-9), and TNF-α at baseline. Baseline measures were compared using Kruskal-Wallis test. In the second phase, to explore oxidative changes during transition from opium use to methadone, blood and urine samples of patients with opioid use disorder were re-evaluated on Days 3, 7, and 14 after methadone therapy. Repeated measures analysis was used to determine the relative contribution of intervention to changes in CAT, GSH, MDA, SOD, MMP-9, and TNF-α level over time.

**Results:** We observed lower SOD and catalase activities, and higher TNF-α and MMP-9 level in patients compared to the two comparison groups. Opioids exacerbated the oxidative imbalance and superimposed the underlying oxidative injury in smoker comparison group. Methadone therapy was associated with lower MMP-9 and TNF-α level, and higher SOD and catalase activities two weeks after therapy; showing an improvement in oxidative profile.

**Conclusion: **This was an investigation indicating an oxidative imbalance before methadone therapy and during early days of transition from opium use to methadone. Being aware of redox status is crucial for determining an appropriate antioxidant therapy in opioid use disorder.

Opioid use disorder is a chronic relapsing impairment, which causes important clinical problems (1). Frequent use of opioids produces reactive oxygen species (ROS) (2-5) and decreases the antioxidant capacity (6), modulating the synaptic plasticity (7, 8) and ultimately contributing to opiate dependence (9). ROS upregulates the inflammatory cytokines and increases matrix metalloproteinase (MMP) activity (10). Previous findings show that morphine treatment leads to MMP-9 activation, which may also contribute to creation of morphine tolerance (11). 

It has been suggested that adjustment of redox (oxidation-reduction) status might be useful in reducing the destructive effects of opioid use disorder (12). 

A variety of opioid agonists including methadone have been prescribed in opioid use disorder (13). A minimum of 190 000 drug-related deaths are reported annually, mostly overdoses and opioid-related (14). Methadone maintenance treatment (MMT) is an important part of medical approach in opioid use disorder (15). Methadone induces receptor endocytosis, reduces opioid tolerance (16), and alters redox activity (17). 

‘Friends for Life’ is a program that can be used for prevention and treatment of anxiety and depression. It induces ROS production through the nicotinamide adenine dinucleotide phosphate (NADH/NADPH) oxidase system (18). Awareness about the redox status is crucial for determining the suitable type, dose, and even timing of antioxidant therapy in opioid use disorder. However, little is known about the oxidative status in patients with opioid use disorder. It is not known whether transition from opium use to methadone improves or exacerbates the oxidative imbalance. The goal of the first phase of the study was to explore the inflammatory and oxidative status of patients with opioid use disorder. We compared plasma oxidative and inflammatory markers of patients with opioid use disorder with those of smoking and non-smoking healthy participants. Furthermore, there is little information about the redox (oxidation-reduction) homeostasis during transition from opium use to methadone. There is also little information about the necessity of antioxidant therapy in this period. The goal of the second phase of the study was to explore oxidative changes during transition from opium use to methadone. Opioids modulate the redox homeostasis. Therefore, understanding the profile of oxidative changes in individuals with opioid use disorder could be significantly beneficial to help select an efficient antioxidant therapy and diminish oxidative damage in clinical settings.

We analyzed catalase (CAT), glutathione (GSH), malondialdehyde (MDA), superoxide dismutase (SOD) activity (as oxidative markers) and also MMP-9 and TNF-α level in the plasma of patients with opioid use disorder during two weeks of methadone therapy.

## Materials and Methods

*Study Participants*

We included 60 individuals (20 smoking, 20 non-smoking healthy persons, and 20 patients with opioid use disorder). Patients with the diagnosis of opioid dependence were recruited from the clinics in the rural community of Noor, in Mazandaran province, Iran. The patients were smokers and their average consumption of cigarette was 1.31 pack per year. They were in induction phase of maintenance treatment of methadone. All the participants were informed about the purpose of the study and signed written consent before entry to the study. The Ethical Board of the Tehran University of Medical Sciences approved the study. Table 1 elucidates the summary of important parameters in controls and patients.

The inclusion criteria were as follow: A minimum of one year of opium use; at least one gram opium consumption; opioid-positive urine rapid test at the first visit; blood pressure at a flat position above 100/65 mmHg; systemic blood pressure under 150 mmHg; diastolic pressure under 90 mmHg; heart rate less than 90 beat per minutes; and no cigarette smoking at least two hours before drawing the blood sample. Opioid dependence was determined through a clinical interview and according to the diagnostic criteria summarized in the Diagnostic and Statistical Manual (DSM-IV); American Psychiatric Association.

The important exclusion criteria were as follow: Previous exposure to methadone; clinical manifestation of chronic liver or renal disease; pregnancy; asthma; cancer; cardiovascular disease; diabetes; rheumatoid arthritis; using vitamin or nutritional supplements in the preceding three months; age under 18 or above 60; and severe weight loss or excessive weight gain within the previous six months. After physical examination and asking some demographic questions, blood and urine samples were obtained. Presence of chronic liver or kidney disease, diabetes, infection, and HIV were evaluated using plasma laboratory tests (Table 1). Methadone was prescribed in the amount of 40 to 80 mg based on physical examination and history of opium use. According to methadone maintenance treatment protocol, 30 to 40 mg methadone was prescribed for the first day of the treatment, and it gradually increased up to 80 mg by assessing the patient’s condition. The mean doses of methadone were approximately 37.5 mg on the third day, 55 mg on the seventh day and 54.5 mg on the 14th day.

All participants completed measures of catalase (CAT), glutathione (GSH), malondialdehyde (MDA), superoxide dismutase (SOD), Metalloproteinase-9 (MMP-9) and Tumor Necrosis Factor-α (TNF-α) at baseline. Urine samples were assessed for methamphetamine, opioids, marijuana, and methadone. Blood samples were centrifuged at 4 000 rpm for 10 minutes and plasma was stored at -70°C for subsequent analysis.

**Figure1:**
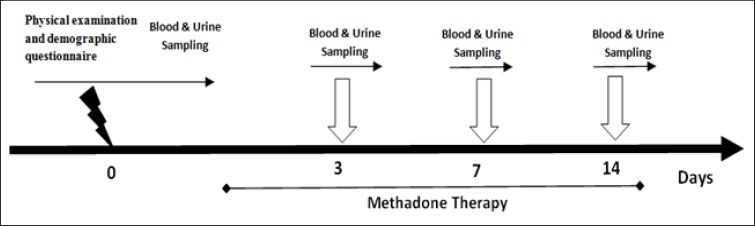
Schematic Representation of Experimental Procedures in Opioid Use Disorder Patients

The goal of the second phase of the study was to explore oxidative changes during transition from opium use to methadone. Blood and urine samples were re-evaluated on Days 3, 7, and 14 after methadone administration (Figure 1). Since opioid metabolites live up to 14 days after use, we evaluated the samples during this period. We obtained blood and urine samples on the first three days and on Days, 7 and 14 after methadone maintenance treatment.

*Statistical Analysis*

SPSS software (Version 19) was used for statistical analysis. All data were expressed as means ± SD. After the Shapiro–Wilk test, baseline measures of the participants were compared using Kruskal-Wallis test. To determine the relative contribution of intervention to changes in CAT, GSH, MDA, SOD, as well as MMP-9 and TNF-α level over time, repeated measures analysis was used to model the within-subject changes over time. Statistical significance was set at p<0.05.

*Measurement of Glutathione*

Total glutathione was measured based on Tietze assay with Griffith’s modifications (19, 20). Briefly, 0.2 mL of fresh blood was mixed with 3 mL precipitating solution. After adding the DTNB (5, 5’-Dithiobis 2-nitrobenzoic acid) solution, the TNB formation was recorded at 412 nm and expressed in mg/dL.

*Measurement of Superoxide Dismutase (SOD) and Catalase Activity*

The erythrocyte SOD activity was quantified by Paoletti assay (21). In this assay, the oxidation of NADPH indicates the presence of superoxide anions. The value was recorded as U/mg Hb. The catalase activity was measured by decomposition of H2O2 and monitoring the decrease in the absorbance of H2O2 at 240 nm (22). Each sample’s value was represented as U/mg Hb.

*Measurement of Malondialdehyde (MDA)*

Plasma MDA level (nM/mL), an index of lipid peroxidation, was assayed in the form of thiobarbituric acid reacting substances (TBARS), and its absorbance was measured at 532 nm as previously defined (23).

Measurement of Metalloproteinase-9 (MMP-9) and Tumor Necrosis Factor-α (TNF-α):

Plasma MMP9 and TNF-α levels were determined using two quantitative sandwich enzyme immunoassay kits (R&D, DTA00C, DMP900, Minneapolis, USA). The monoclonal antibody of human MMP9 or TNF-α were precoated onto two microplates. The samples and standards were poured into the wells of microplates. After washing and adding the substrate solution, color development was recorded at 450 nm, with the correction set at 540 nm using a microplate reader. 

*Biochemical Measurements*

Blood concentrations of creatinine, BUN, alanine aminotransferase (ALT), and aspartate aminotransferase (AST) were measured using spectrophotometry. The hemoglobin was measured by photometric assay at 540 nm.

## Results

**Figure 2:**
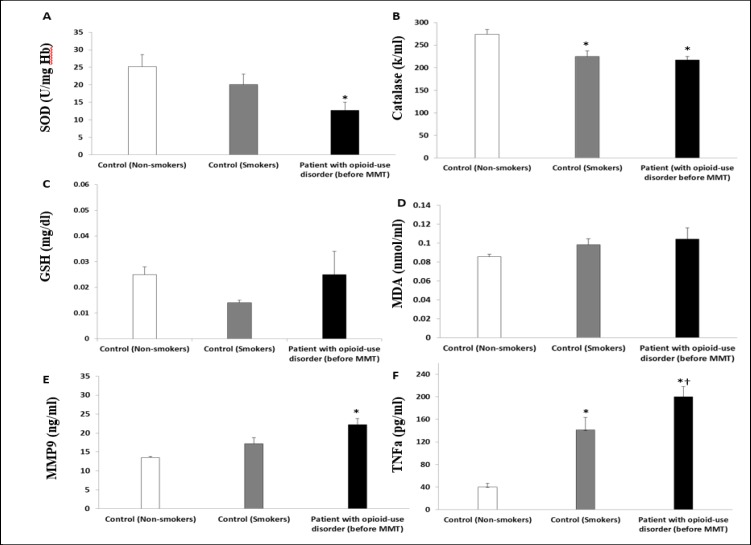
Comparison of Erythrocyte Superoxide Dismutase (A), Catalase Activity (B), Glutathione (C), Plasma Malondialdehyde, MDA (D), Plasma MMP9 (E), Plasma TNF-α (F) in Non-smokers, Smokers and Patients with Opioids Use Disorder (before MMT). The Data Are Presented as Means ± SD. *P<0.05 Compared to the Non-smoker Comparison Group. †P<0.05 Compared to the Smoker Comparison Group

Results of the first phase of the study presented in Table 2 and are as follow: 

Comparison of Plasma Oxidative and Inflammatory Markers in Patients with Opioid Use Disorder (Before Methadone Therapy) with Smoking and Non-smoking Healthy Participants:

Figure 2A demonstrates erythrocyte superoxide dismutase (SOD) activity. There was lower SOD activity in patients before methadone therapy (12.71±10.005, U/mg Hb, p<0.05) compared to the smoker (20.08±10.34) or non-smoker (25.18±11.25) comparison groups. Catalase activity is presented in Figure 2B, demonstrating a lower catalase activity in the smoker group (224.56±37.7 k/mL) and in patients with opioid use disorder (216.82±33.4 k/mL) compared to the non-smoker (274.22±31.6 k/mL, p<0.05, Figure 2B) group.

However, no significant difference was found between non-smoker and smoker comparison groups (0.025±0.009 vs. 0.014± 0.005 mg/dL, p>0.05, Figure 2C) in the erythrocyte GSH level. No significant difference was found between comparison groups and patients with opioid use disorder before methadone treatment (0.025±0.04 vs. 0.025± 0.009 mg/dL, p>0.05). Also, the MDA level was not significantly different between comparison groups and patients with opioid use disorder before methadone therapy (0.085±0.007 vs. 0.104±0.05 nm/mL, p>0.05, Figure 2D).

Figure 2E demonstrates higher plasma MMP9 in patients with opioid use disorder before methadone therapy (22.21±6.32 ng/mL, p<0.05) compared to the smoker (17.21±6.80) or non-smoker (13.47±1.35) comparison groups. Furthermore, a significant higher plasma TNF-α was detected in the smoker group compared with the non-smoker group (141.23±96.2 vs. 40.22±25.8 pg/mL, p<0.05, Figure 2F). The plasma TNF-α level in patients with opioid use disorder was higher (199.96±69.14 pg/mL, p<0.05) compared to the smoker or non-smoker comparison groups.

Results of the second phase of the study are as follow:

Effect of Intervention on Oxidative and Inflammatory Markers in Patients with Opioid Use Disorder 3, 7, and 14 Days after Methadone Therapy

**Figure 3:**
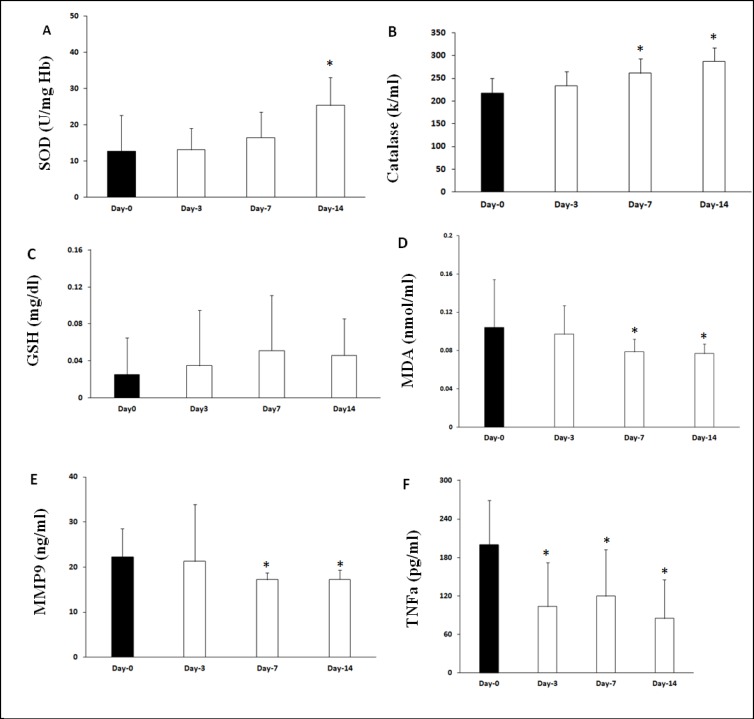
Comparison of Oxidative and Inflammatory Markers, 3, 7 and 14 Days after Methadone Therapy in Patients with Opioid Use Disorder. The Erythrocyte SOD (A) and Catalase Activity (B) Were Significantly Higher on Day 14 after Methadone Therapy. The Effect of MMT on GSH (C) and MDA (D) levels; as Shown, lower MDA level was Observed on Days 7 and 14 after MMT. The Plasma level of MMP-9 (E) and TNF-α (F) was lower on Days 7 and 14 after MMT. Data Are Expressed as Means ± SD. *P<0.05 Compared with Day-0 (before MMT)

A significant effect of time was found for SOD measures. Linear mixed model analysis found the higher SOD activity on day 14 (β=12.67, CI 8.25-17.08; p<0.001). Figure 3A shows that on Days 3 (13.15 ± 5.9, U/mg Hb, p>0.05) and 7 (16.5 ± 6.9) after methadone therapy, there was no significant change in SOD activity, when compared with before treatment. However, a significant higher SOD activity was observed on Day 14 (25.38 ± 7.6, U/mg Hb, p < 0.05) compared to before treatment.

Moreover, in catalase results, a significant effect of time was found for CAT measures. Compared to baseline, patients had improved CAT levels at Days 3, 7, and 14. Linear mixed model analysis found that CAT increased from baseline to Days 3 (β=18.90, CI 5.07-32.72; P=0.01), 7 (β=43.64, CI 23.68-63.59; P<0.001), and 14 (β=69.24, CI 51.34-87.13; p<0.001). Catalase was higher on Days 3 (234 ± 31.45, p>0.05), 7 (261.3 ± 31.68 k/mL, p<0.05), and 14 (287.11 ± 29.89 k/mL, p<0.05, Figure 3 B), when compared with before methadone therapy. However, the level of glutathione did not show any significant changes on Days 3 (0.0346 ± 0.06), 7 (0.051 ± 0.06), or 14 (0.0459 ± 0.04, mg/dL, p>0.05) after methadone treatment, when compared with before methadone therapy (Figure 3C).

Furthermore, linear mixed model analysis revealed that MDA was significantly decreased from baseline on Day 7 (β=-0.01, CI -0.03-2.6; P=0.050), and 14 (β=-0.02, CI -0.04-0.08; P=0.008). MDA was significantly decreased on Days 7 (0.078 ± 0.013 vs. 0.104 ± 0.05 nmol/mL, p<0.05, Figure 3D) and 14 (0.076 ± 0.01) after methadone therapy, compared to before methadone therapy (0.078 ± 0.013 vs. 0.104 ± 0.05 nmol/mL, p<0.05, Figure 3D). 

Significantly lower plasma MMP9 levels were found on Days 7 and 14 after methadone therapy compared with before methadone therapy (17.24 ± 0.56 vs. 22.21 ± 1.62 ng/mL, p<0.05, Figure 3E). Approximately, a similar pattern was noted for the plasma TNF-α level. Lower TNF-α level was seen on Days 3 (103.69 ± 18.34), 7 (119.55 ± 19.5), and 14 (85.05 ± 16.02 vs 199.96 ± 18.47, pg/mL, p<0.05, Figure 3F) after methadone therapy compared with before methadone therapy. 

**Table 1:** Demographic Characteristics of the Subjects

**Factors**	**Patients with Opioid Use ** **Disorder**	**Smoking Comparison ** **Group**	**Non-smoking ** **Comparison Group**
Age (years)	36.2±8.72	35.3±8.25	35.35±10.15
Sex (% male)	100	100	100
Use period (year)	9.55±6.5	-	-
Use amount (gr)	2.85±1.44	-	-
Opioid-positive urine test (%)	100	0	0
Route of administration (% smoking/oral)	60/40	-	-
BMI (kg/m2)	25.71±4.12	26.5±4.04	26.43±4.12
Hb (g/dL)	15.085	15.46	15.32
Cigarette use period (year)	14.5±6.81	11.15±6.76	-
Cigarette use amount (pack per year)	1.31	1.1	-
HIV	Negative	Negative	Negative
HBS	Negative	Negative	Negative
BUN(mg/dL)	12.9±0.67	14.7±.0.63	14.2±0.65
Creatinine (mg/dL)	0.74±0.02	0.86±0.04	0.84±0.04
AST IU/L	23.81±.6	26.9±1.1	24.4±1.5
ALT IU/L	24.2±1.9	28.6±1.6	24±1.7

Data are mean±SD. There was no significant difference between three groups in demographic and baseline characteristics. ALT, Alanine aminotransferase; AST, Aspartate aminotransferase; BMI, Body mass index; BUN, Blood urea nitrogen; HBS, Hepatitis B surface antigen; HIV, The human immunodeficiency virus.

**Table 2:** Comparison of Erythrocyte Superoxide Dismutase, Catalase Activity, Glutathione, Plasma Malondialdehyde, MMP9 and TNF-α in Non-smokers, Smokers and Patients with Opioids Use Disorder **(before MMT)**

**Groups**	**SOD** **(U/mg Hb)**	**CAT** **(k/mL)**	**GSH** **(mg/dl)**	**MDA** **(nM/mL)**	**MMP-9** **(ng/mL)**	**TNF-** **α** **(pg/mL)**
Non-smokers	25.18±11.25	274.22±31.6	0.014± 0.005	0.085±0.007	13.47±1.356	40.22±25.8
Smokers	20.08±10.34	216.82±33.4*	0.0252±0.003	0.095±0.022	17.21±6.803	141.23±96.2*
Patients with opioid use disorder (before MMT)	12.71±10.005*	224.56±37.7*	0.0253± 0.009	0.10±0.05	22.21±6.32*	199.96±69*,†

The data are presented as means ± SD.

* P<0.05 compared to the non-smoker comparison group.

† P<0.05 compared to the smoker comparison group. Catalase (CAT), Glutathione (GSH), Malondialdehyde (MDA), Superoxide dismutase (SOD), Metalloproteinase-9 (MMP-9) and Tumor Necrosis Factor-α (TNF-α).

## Discussion

In the first phase of the present study, it was initially necessary to determine the oxidative status in patients with opioid use disorder to find any differences between them and the comparison groups. We found an oxidative imbalance in patients with opioid use disorder before methadone therapy as demonstrated by lower SOD and catalase activity compared to the non-smoker comparison group.

Consistent with these results, it has been shown that opioid administration decreases the levels of catalase and SOD activity (24). Superoxide dismutase (SOD) serves as the first line of antioxidant defense against ROS (25). In the present study, no significant alteration was detected in GSH, which is among the second line of antioxidant defenses that are influenced by several compensatory mechanisms (12). As a result of this 

Complex relationship among non-enzymatic antioxidants, GSH levels did not show any significant change.

Patients with opioid use disorder were carefully selected according to the inclusion and exclusion criteria. Several cases were lost to follow-up due to their unavailability for repeating the urine and blood sample. We evaluated the oxidative status during transition from opium use to methadone. It seems that the transition time from opium use to methadone is a more vulnerable phase, which may need antioxidant intervention.

Furthermore, patients left the study before completion due to several reasons including absences in sampling days, positive urine tests, no tendency for continuing the study, and illness. Opium use from the beginning of methadone therapy was one of our exclusion factors. Therefore, we excluded positive urine rapid tests. Hence, small sample size was a limitation in our study. Any case with methamphetamine or positive urine tests was excluded during the study.

Cigarette smoking is a serious source of oxidative stress (27), and chronic smoking induces the release of proinflammatory markers, such as TNF-α (27) MMP9, which is a marker of inflammation and increases with smoking (28). Since all the patients with opioid use disorder were smokers as well, we needed to include a smoker comparison group in our design. There was an oxidative imbalance in the smoker group as shown by lower SOD activity compared to the non-smoker comparison group. There was also higher MMP9 and TNF-α level in the smoker group compared to the non-smoker group. However, we also observed a significant difference between patients with opioid use disorder before methadone therapy compared to the smoker comparison group. This implies that opioids exacerbate the oxidative imbalance and superimpose the underlying oxidative injury. Moreover, the impact of individual smoking manner cannot be excluded. We established a period of abstaining from cigarettes for at least two hours before blood sampling. We also tried our best to limit the nutritional confounding factors by recruiting individuals from a narrow geographic area and with similar nutritional habits. Naturally, other confounding factors cannot be ignored.

In the second phase of the study, to clarify the oxidative changes during transition from opium use to methadone, we compared the data of patients with opioid use disorder before and after methadone therapy. Any case with methamphetamine or opioids-positive urine tests was excluded during the study. The range of the methadone dose was 40 to 80 mg and prescribed based on physical examination and history of opium use. Generally, 30 to 40 mg doses of methadone avoid most withdrawal symptoms and craving (29). The mean dose of methadone was 37.5 mg on the third day, 55mg on the seventh day, and approximately 54.5 mg on the 14th day. The increase of methadone dose was based on withdrawal symptoms and protocols directions.

We found improvement in oxidative imbalance 14 days after methadone therapy as shown by higher SOD and catalase activity compared to before methadone therapy. Moreover, we observed lower MMP9 and TNF-α level on Days 7 and 14 after methadone therapy, implying diminished inflammation. This effect, at least in part, may be related to the phospholipase D2 (PLD2) activation by methadone. Methadone produces low concentration of ROS via the NADH/NADPH oxidase pathway, which is necessary for µ-opioid receptor endocytosis (18). It has been shown that such opioids as methadone with the ability of PLD2 activation are less neurotoxic than such opioids (as morphine that cannot activate PLD2 (30). The release of endogenous opioids following stress or pain may be protective via the activation of PLD2 and subsequently low ROS generation, which ensures appropriate cell responses to different stimuli (31). On the other hand, the result also suggests that there may be a necessity for an antioxidant therapy at the first week of the shift from opium use to methadone therapy. Although the replenishment of oxidative homeostasis should alleviate the destructive effects of opioid use disorder (12), the majority of published clinical trials using antioxidants have failed to demonstrate positive outcomes (32). To have a successful antioxidant therapy, the pattern of changes in oxidative markers should be revealed and used as criteria in selecting the defense strategy against oxidative stress (33). This pattern of changes determines the efficient type, dosage, and even timing of the antioxidant therapy.

## Limitations

The opium use from the beginning of methadone therapy was one of our exclusion factors. Therefore, we excluded positive urine rapid tests. Hence, the small sample size was a limitation in our study. Any case with methamphetamine or positive urine tests was excluded during study.

## Conclusion

The results of the first phase of the study indicated an oxidative imbalance in the smoker group and revealed that opioids exacerbate the oxidative imbalance and superimpose the underlying oxidative injury. In the second phase of the study, we found an oxidative imbalance in patients with opioid use disorder before methadone therapy and during early days of transition from opium use to methadone, which should be considered in therapeutic protocol of patients with opioid use disorder. Being aware of oxidation status is critical for determining the efficient antioxidant therapy in opioid use disorder.
